# Proteomic analysis of human vitreous humor

**DOI:** 10.1186/1559-0275-11-29

**Published:** 2014-07-14

**Authors:** Krishna R Murthy, Renu Goel, Yashwanth Subbannayya, Harrys KC Jacob, Praveen R Murthy, Srikanth Srinivas Manda, Arun H Patil, Rakesh Sharma, Nandini A Sahasrabuddhe, Arun Parashar, Bipin G Nair, Venkatarangaiah Krishna, TS Keshava Prasad, Harsha Gowda, Akhilesh Pandey

**Affiliations:** 1 Institute of Bioinformatics, International Technology Park, Bangalore 560 066, India; 2 Amrita School of Biotechnology, Amrita Vishwa Vidyapeetham, Kollam, Kerala 690 525, India; 3 Vittala International Institute Of Ophthalmology, Bangalore, Karnataka 560085, India; 4 Department of Biotechnology, Kuvempu University, Shankaraghatta, Karnataka 577 451, India; 5 Centre of Excellence in Bioinformatics, Bioinformatics Centre, School of Life Sciences, Pondicherry University, Puducherry 605 014, India; 6 Department of Neurochemistry, National Institute of Mental Health and Neuro Sciences, Bangalore 560 006, India; 7 Armed Forces Medical College, Pune 411 040, India; 8 Department of Biological Chemistry, McKusick-Nathans Institute of Genetic Medicine, Johns Hopkins University School of Medicine, Baltimore 21205 MD, USA; 9 Department of Oncology and Pathology, Johns Hopkins University School of Medicine, Baltimore 21205 MD, USA

**Keywords:** Retina, SCX chromatography, OFFGEL electrophoresis, Proteome discoverer, Secreted proteins, Protein biomarkers, Body fluid proteomics

## Abstract

**Background:**

The vitreous humor is a transparent, gelatinous mass whose main constituent is water. It plays an important role in providing metabolic nutrient requirements of the lens, coordinating eye growth and providing support to the retina. It is in close proximity to the retina and reflects many of the changes occurring in this tissue. The biochemical changes occurring in the vitreous could provide a better understanding about the pathophysiological processes that occur in vitreoretinopathy. In this study, we investigated the proteome of normal human vitreous humor using high resolution Fourier transform mass spectrometry.

**Results:**

The vitreous humor was subjected to multiple fractionation techniques followed by LC-MS/MS analysis. We identified 1,205 proteins, 682 of which have not been described previously in the vitreous humor. Most proteins were localized to the extracellular space (24%), cytoplasm (20%) or plasma membrane (14%). Classification based on molecular function showed that 27% had catalytic activity, 10% structural activity, 10% binding activity, 4% cell and 4% transporter activity. Categorization for biological processes showed 28% participate in metabolism, 20% in cell communication and 13% in cell growth. The data have been deposited to the ProteomeXchange with identifier PXD000957.

**Conclusion:**

This large catalog of vitreous proteins should facilitate biomedical research into pathological conditions of the eye including diabetic retinopathy, retinal detachment and cataract.

## Background

The vitreous is a highly hydrated gelatinous mass that fills the space between the lens and the retina. The vitreous is adherent to the retina diffusely though the adhesion is strongest at the anterior border of retina, the macula, the optic nerve head, over lattice degenerations and areas of scars. The major function of the vitreous is to allow light to reach the retina and maintain the shape of the eyeball. The formation of vitreous occurs in two phases. Primary vitreous is formed by the third or fourth week of gestation, when the neural ectoderm separates from the surface ectoderm. The space between the two is the future vitreous cavity. This space is bridged by fibrillar material which is thought to be collagenous in nature. By the time the fetus reaches the 10 mm stage, mesodermal cells enter the vitreous space via the fetal fissure and develop into hyaloid vessels which branch throughout the vitreous cavity [1]. In the adventitia surrounding the vessels, there are mononuclear phagocytes and fibroblasts which are thought to later differentiate into hyalocytes. This cellular vitreous is the primary vitreous. Acellular structures begin to appear by end of the sixth week of gestation between the retina and the hyaloid vasculature. This secondary vitreous is essentially extracellular matrix consisting primarily of type 2 collagen [2]. With the development of the secondary vitreous, the hyaloid vascular system regresses. Hyalocytes appear to be the most important cells and after birth, there is no new migration of these cells into the vitreous cortex. Thus, with an increase in globe size and vitreous cortex surface area, there is a decrease in the density of hyalocytes. Since the vitreous acts as a metabolic repository for the retina, hyalocytes and surrounding tissues [3], some of the proteins in vitreous humor could be contributed by these surrounding tissues. Its viscosity is two to four times greater than water, which gives it a gelatinous consistency [4]. The human vitreous is composed of a complex network of cross-linked collagen fibres of types II, V, IX and XI of which type II is the most abundant. Non-collagenous structural proteins are less abundant as compared to collagen fibrils. Hyaluronic acid, a glycosaminoglycan is also found in abundance in the vitreous [5,6]. A significant amount of prealbumin and transferrin in the vitreous has also been reported [7-9]. Of the soluble proteins that constitute the vitreous, sialic acid containing glycoproteins constitute the largest fraction [10].

It is well understood that the changes occurring in the retina are closely linked to biochemical changes occurring in the vitreous humor [11]. Vitreous humor does not have any blood vessels but is nourished by vessels of the retina and the ciliary body. As the vitreous can be easily obtained during vitrectomy surgery, studying the changes occurring in the vitreous could provide valuable information about pathological changes occurring in the retina in disorders of the eye. Many researchers have studied the proteins of the normal vitreous using different techniques and some of them have also catalogued various proteins [12-16]. Nakanishi *et al.*, have catalogued 51 proteins in the vitreous by Matrix assisted laser desorption ionization-time of flight (MALDI-TOF) mass spectrometry [12]. Kim *et al.* employed immunoaffinity depletion followed by nano-liquid chromatography-Matrix assisted laser desorption ionization (LC-MALDI-MS/MS) resulting in the identification of 346 proteins from non-diabetic controls [13]. In a subsequent study, Gao *et al*. identified 252 proteins from the vitreous employing sodium dodecyl sulphate polyacylamide gel electrophoresis (SDS-PAGE) analyzed by LC-MS/MS [14]. Aretz et al have identified 1,111 distinct proteins by employing different protein prefractionation strategies such as liquid phase isoelectric focussing, 1D SDS gel electrophoresis and a combination of both [16].

Here, we identified 1,205 proteins of which 682 proteins have been detected in the vitreous humor for the first time. A comprehensive proteomic profiling of normal vitreous would serve as an invaluable template for future studies that focus on protein dynamics in vitreous in pathological conditions.

## Results and discussion

**Figure 1:**
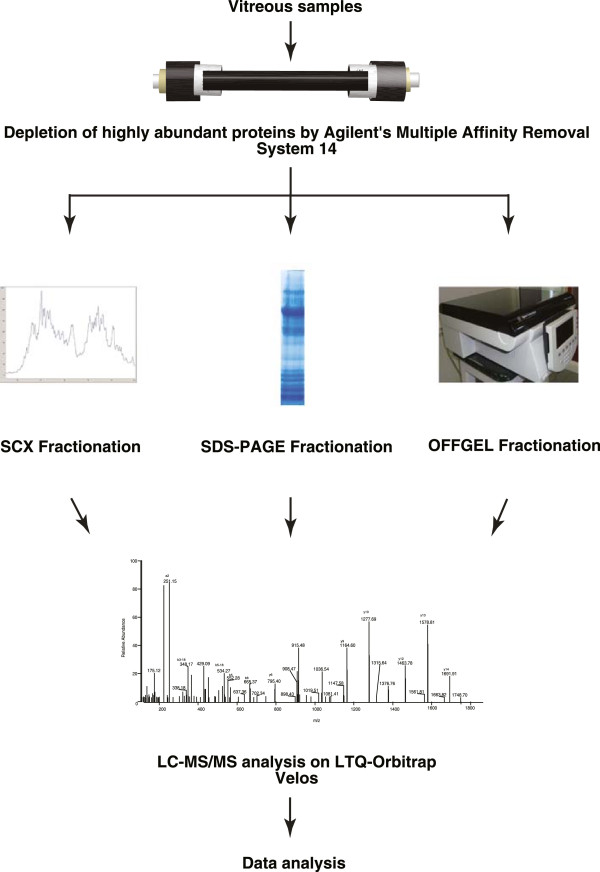
**Experimental design for proteomic characterization of vitreous humor.** Pooled vitreous humor samples were depleted of abundant proteins using Agilent’s MARS 14 column followed by *in-gel* digestion, *in-solution* digestion and OFFGEL electrophoresis. The samples were analyzed on LTQ-Orbitrap Velos mass spectrometer.

Proteomic analysis of the vitreous humor was carried out by depleting the samples of abundant proteins and subjecting it to multiple fractionation techniques, such as In-gel digestion, SCX and OFFGEL fractionation. LC-MS/MS analyses of the different fractions were carried out on an LTQ-Orbitrap Velos (Thermo Electron, Bremen, Germany) mass spectrometer. The corresponding MS data were searched using the search algorithms Mascot, SEQUEST and X! Tandem against NCBI RefSeq 59 human protein database containing 36,211 sequences with known contaminants (Figure 1). This resulted in the identification of 7,642 peptides from 1,205 proteins. A complete list of proteins identified in vitreous is provided in Additional file 1: Table S1 along with their known sub cellular localization, molecular function, biological process, domains/motifs, number of peptides and sequence coverage. Protein identifications include 41 proteins designated as ‘missing proteins’ by c-HPP and these are highlighted in Additional file 1: Table S1. A list of peptides identified from this study from all three searches is provided in Additional file 2: Table S2. When compared to previous reports, 682 proteins out of 1,205 were found to be uniquely identified in this study. A partial list of these proteins is provided in Table 1. The description of 3 novel proteins identified in this study and four previously reported proteins is provided in following sections.

**Table 1:** A partial list of novel proteins identified in this study

**Gene symbol**	**Description**	**Cellular component**	**Biological process**	**Molecular function**
*LCN1*	Lipocalin-1	Extracellular	Transport	Transporter activity
*TIMP1*	Metalloproteinase inhibitor 1	Extracellular	Cell growth	Extracellular matrix structural constituent
*SPARC*	Sparc	Extracellular	Cell growth and/or maintenance	Extracellular matrix structural constituent
*CHRDL1*	Chordin-like protein 1	Unknown	Cell communication, Signal transduction	Molecular function unknown
*DCD*	Dermcidin	Extracellular	Immune response	Molecular function unknown
*C19orf10*	Hypothetical protein loc56005	Unknown	Cell communication, Signal transduction	Growth factor activity
*PI3*	Elafin	Extracellular	Protein metabolism	Protease inhibitor activity
*ADIPOQ*	Adiponectin	Extracellular	Metabolism, Energy pathways	Molecular function unknown
*SFRP2*	Secreted frizzled-related protein 2	Extracellular	Cell communication, Signal transduction	Molecular function unknown
*PTX3*	Pentraxin-related protein	Extracellular	Immune response	Defense/immunity protein activity
*PTPMT1*	Protein-tyrosine phosphatase mitochondrial 1	Unknown	Cell communication, Signal transduction	Protein tyrosine/serine/threonine phosphatase activity
*FLG*	Filaggrin	Nucleus	Cell communication, Signal transduction	Calcium ion binding
*ACTB*	Actin, cytoplasmic 1	Cytoplasm	Cell growth and/or maintenance	Structural constituent of cytoskeleton
*AZU1*	Azurocidin	Cytoplasmic vesicle	Immune response	Defense/immunity protein activity
*NENF*	Neudesin	Extracellular	Biological_process unknown	Kinase regulator activity

Proteomic analysis of the vitreous has been carried out by many groups using different techniques as summarized in Table 2. Some of the proteins not identified in the present study in comparison to previous studies may be due to differences in sample preparation methods or mass spectrometry platforms employed. Since obtaining normal vitreous from healthy individuals is not a possibility, clinically normal appearing vitreous samples from patients undergoing vitrectomy surgery for macular hole or traumatic cataract and cataract surgery for congenital cataract were pooled for the analysis. There is a potential possibility that the vitreous proteome in some cases is influenced by associated pathology in these subjects. However, this was close to normal vitreous that we could obtain for the study.

**Table 2:** A summary of some of the previously published proteomics studies on vitreous humor and the corresponding mass spectrometry platforms

	**Study**	**Fractionation/separation method**	**Mass spectrometer**	**Number of of proteins identified**
1.	Aretz S *et al.,*[16]	SDS-PAGE, Liquid phase IEF	LTQ-ORBITRAP XL	1,111
2.	Simo *et al*., [17]	DIGE, Western blot	MALDI	2
3.	Gao *et al*. [14]	SDS-PAGE	LTQ	252
4.	Kim *et al*. [13]	2-DE	(IS)/2-DE/MALDI-MS, LC-MALDI-	346
		SDS-PAGE	MS/MS, and LC-ESI-MS/MS	
5.	Ouchi *et al*. [15]	2D-PAGE	CapLC system with QTOF2	15
6.	Wu *et al*., [18]	SDS-PAGE	MALDI-TOF	12
7.	Yamane *et al*., [19]	2D-PAGE	Q-TOF	18
8.	Nakanishi *et al*. [12]	2D-PAGE	MALDI-TOF	51
			LCQ DECA	

### Known proteins in the vitreous humor

**Figure 2:**
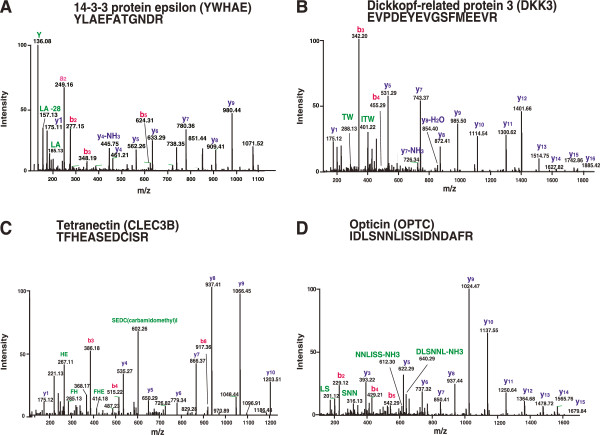
**Representative MS/MS spectra of known proteins in vitreous that were identified in this study. A**. peptide YLAEFATGNDR belongs to 14-3-3 protein epsilon. **B**. Peptide EVPDEYEVGSFMEEVR belongs to dickkopf-related protein 3. **C**. Peptide TFHEASEDCISR belongs to tetranectin. **D**. Peptide IDLSNNLISSIDNDAFR belongs to opticin.

Among the identified proteins in vitreous, we found many proteins that had been previously described in the vitreous, confirming the validity of the proteomic approach undertaken by us. Among the proteins previously reported in vitreous are aldolase A, fructose-bisphosphate (ALDOA), cystatin C (CST3), dickkopf homolog 3 (DKK3), orosomucoid 2 (ORM2), serpin peptidase inhibitor, clade C member 1 (SERPINC1), insulin-like growth factor binding protein 7 (IGFBP7), lipocalin 2 (LCN2), TIMP metallopeptidase inhibitor 2 (TIMP2), C-type lectin domain family 3, member B (CLEC3B), lectin mannose-binding 2 (LMAN2), opticin (OPTC), serpin peptidase inhibitor clade F (SERPINF1). Representative MS/MS spectra of some identified proteins in this study are shown in (Figure 2).

We identified several components of complement system including complement C1q subcomponent subunit A (C1QA), C1QB, C1QC, C1QL3, C1QTNF3, C1QTNF5, C1R, C1RL, C1S, C2, C3, C4A, C4B, C4BPA, C5, C6, C7, C8A, C8B, C8G, C9, CFB, CFD, CFH, CFHR1, CFHR2, CFHR3, CFI.

Activation of the complement pathways can initiate and accelerate thrombosis, apoptosis and leukostasis, all of which could be important steps in the development of diabetic retinopathy [20]. Some of the complement factors are also associated with age-related macular degeneration (AMD). Raychaudhuri et al., have shown that compromised complement factor H (CFH) function contributes to pathogenesis of AMD [21]. CFH risk allele reveals a polymorphism representing a tyrosine to histidine change at amino acid 402, which is associated with AMD [22]. This region of CFH is crucial for binding of heparin and C-reactive protein [23]. Ennis et al., have also shown that SNP in complement factor 1 is associated with risk of AMD [24].

Opticin (OPTC) is a member of the small leucine-rich repeat protein (SLRP) family. Opticin is expressed in the retina, skin, iris, vitreous humor, non-pigmented epithelium of the ciliary body, sclera, optic nerve, choroid, corneal epithelium, uveal tract and lens [25-27]. It is associated with age related macular degeneration and posterior column ataxia with retinitis pigmentosa, both of which are inherited eye diseases [28].

Retinol-binding protein 3 (RBP3) is a soluble single subunit glycoprotein that is synthesized and secreted by rod photoreceptor cells into the interphotoreceptor matrix [29-32]. It is believed to transport all trans retinol to the retinal pigment epithelium (RPE) and 11-cis retinal from the RPE to the bleached photoreceptors, thus playing an important role in the visual cycle [33]. A mutation in the RBP3 gene (Asp1080Asn) has been linked to autosomal recessive retinitis pigmentosa [34]. RBP3 is known to modulate the notch signal transduction by interacting with the phosphorylated intracellular domain of the notch receptor [35]. It is surprising that molecules such as RBP3, which appears to function at the back of the retina farthest from the vitreous, could still be found in the vitreous. The possible explanation for this could be that even though the intravascular contents are prevented from directly reaching the vitreous by the presence of the blood ocular barrier, there is no evidence of an effective barrier for proteins in the intercellular and interstitial spaces of retina and surrounding tissues.

Serpin peptidase inhibitor, clade F, member 1 (SERPINF1) is a 50 kDa secreted glycoprotein and reported to be expressed in many tissue and vitreous [36,37]. It belongs to a group of serine protease inhibitors with anti-angiogenic activities [38,39]. SERPINF1 concentration is found to be significantly lower in the vitreous fluid of subjects with PDR [40-42].

### Novel proteins identified in the vitreous humor

**Figure 3:**
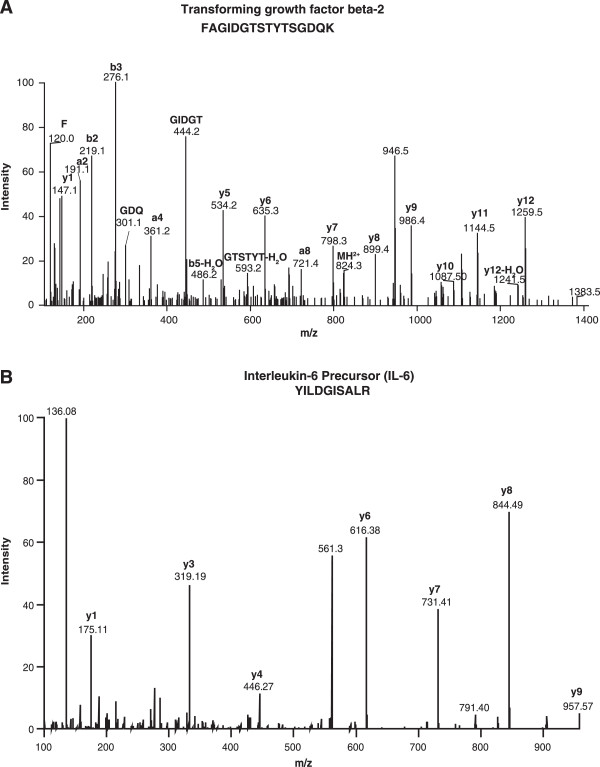
**Representative MS/MS spectra of novel proteins identified in this study. A**. Peptide FAGIDGTSTYTSGDQK belongs to Transforming growth factor beta-2 (TGFB2). **B**. Peptide YILDGISALR belongs to Interleukin-6 Precursor (IL-6).

Representative MS/MS spectra of two unique proteins identified in this study –Interleukin 6 (IL-6) and transforming growth factor beta-2 (TGFB2) are shown in Figure 3. Interleukin-6 is a cytokine released in acute and chronic inflammation. This molecule has been extensively studied as an early indicator of inflammatory disorders such as acute pancreatitis and urticaria [43,44]. All insulin-like growth factor-binding proteins (IGFBP) are secreted and have a leader sequence. They are conserved at amino and carboxyl terminal with 18 cysteine residues. All these genes encode proteins with an IGFBP domain and a thyroglobulin type-I domain. These proteins bind to insulin-like growth factors (IGFs) I and II and are found in the plasma in the form of glycosylated and non-glycosylated forms [45-48]. IGFBP4, IGFBP6 are O-glycosylated and IGFBP5 and IGFBP6 are N-glycosylated [49,50]. IGFBP6 is tumor suppressor, [51] extracellular protein [52] and preferentially binds to IGF-II [53]. IGFBP2 serves as an antiapoptotic biomarker [54]. We identified several members of the insulin-like growth factor-binding protein (IGFBP) family including IGFBP2, IGFBP4, insulin-like growth factor 2 (IGF2), insulin-like growth factor binding protein acid labile subunit (IGFALS). Other members of this family, IGFBP5, IGFBP6 and IGFBP7, have already been identified in previous studies.

We compared our results with other high throughput studies on vitreous humour [13,14,16]. We have identified 523 proteins identified by other studies. In addition, we have identified 682 novel proteins not described in previous studies. Of the 682 novel proteins, we identified proteins such as S-Arrestin(SAG) and transforming growth factor-beta 2 (TGF-B2) which are involved in the pathophysiology of ocular diseases. S Arrestin, also known as S antigen(S-Ag) is a major photoreceptor protein. It is a member of the beta-arrestin protein family which participate in agonist-mediated desensitization of G-protein-coupled receptors. Arrestin preferentially binds light activated phosphorylated rhodopsin and prevents further signaling by direct competition with transducin, a visual G-protein [55]. It is expressed in the retina and pineal gland. This protein is highly antigenic and has been implicated in experimental uveoretinitis. There is also recent evidence to suggest S-Ag specific T cells may be involved in the pathophysiology of Bechets disease, a chronic, relapsing, multisystem inflammatory disorder characterized by recurrent oral and genital ulcers, severe intraocular inflammation and skin lesions [56]. Mutations in the S Arrestin gene is also associated with an autosomal recessive form of night blindness known as Oguchi disease [57-59].

Transforming growth factor-beta 2(TGF-B2) belongs to the TGFB family of cytokines. These proteins bind to their transmembrane receptors, which in turn activate their downstream effectors like SMAD proteins which are known to regulate cell proliferation, apoptosis and differentiation [60]. Primary open angle glaucoma is a disease of the eye which is characterized by elevated intra ocular pressure due to increased resistance to the outflow of aqueous humor through the trabecular meshwork. TGF-B2 has been shown to be elevated in the aqueous humor of patients with primary open angle glaucoma. Its role in glaucoma is thought to be due to the increased production of extracellular matrix in the trabecular meshwork [61]. A genetic defect in the gene that codes for this protein is associated with Peter's anomaly which is a congenital defect of the anterior chamber of the eye [62].

### Gene ontology analysis

**Figure 4:**
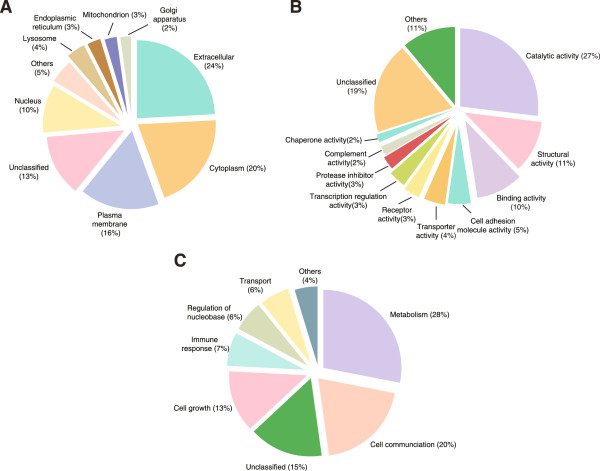
**Subcellular localization and functional annotation of proteins identified in vitreous humor. A**. Gene Ontology analysis for subcellular localization of identified proteins. **B**. Molecular functions of identified proteins. **C**. Biological processes of the identified proteins. The data regarding proteins was obtained from Human Protein Reference Database (HPRD: http://www.hprd.org).

We carried out a bioinformatics analysis of subcellular localization, molecular function and biological processes by searching the identified proteins against the manually curated Human Protein Reference Database (HPRD; http://hprd.org) and Human Proteinpedia (http://humanproteinpedia.org) [63-65]. Of the 1,205 proteins identified in this study, 599 proteins possess signal peptides and 318 proteins are reported to be localized in extracellular compartment. As illustrated in Figure 4A, the majority of the proteins reported in our study were localized to extracellular space (24%), cytoplasm (20%) or plasma membrane (14%). We also classified proteins based on molecular functions and biological processes as shown in Figure 4B, C. Classification based on molecular function showed that the large majority of the proteins are involved in catalytic activity (27%), structural activity (10%), binding activity such as calcium ion binding, receptor binding and complement binding (10%), cell adhesion molecule activity (4%), and transporter activity (4%). A large group (19%) of proteins are still unclassified in terms of their molecular function. Further categorization was done for biological processes which constitute metabolism (28%), cell communication (19%), cell growth (12%), and immune response (7%).

## Conclusions

Our study provides a comprehensive proteomics profile of the human vitreous humor. Many of the pathologic changes occurring in the retina are likely to be reflected in the vitreous because of its close proximity to the retina and also because of the breakdown of the blood retinal barrier. Hence, a proteomic study of the vitreous in related retinal diseases such as diabetic retinopathy, retinal detachment and central or branch retinal vein occlusions would provide valuable insights about the pathophysiology of these diseases. The information from our study could serve as a baseline for future studies especially those aimed at identifying biomarkers for retinal disorders.

## Materials & methods

### Vitreous sample collection

The vitreous samples for the proteomic analysis were obtained from five patients undergoing vitrectomy for macular hole, three patients with congenital cataract who underwent cataract surgery with intra-ocular lens implantation and primary posterior capsulotomy and two samples from patients with traumatic cataract with undisturbed vitreous and intact lens capsule but who also needed vitreous surgery due to zonular damage. All samples were collected by pars plana vitrectomy, were centrifuged at 13,000 rpm at 4°C for 15 minutes and archived at −80°C until further use. Informed consent was obtained from all the subjects and the research adhered to the tenets of Declaration of Helsinki. The study was approved by the Ethics committee “Science for Health” under the approval number 20080713/SFH-014/010. Pooled samples were concentrated by 3 kDa filter through low-adsorption membranes (Amicon, Milliore, Billerica, MA). Protein estimation was carried out for pooled concentrated sample using Lowry’s assay (BioRad Laboratories). A total of 4 mg protein was subjected to Agilent’s Multiple Affinity Removal System 14 (MARS 14) for depletion of abundant proteins. MARS 14 column is routinely used for depletion of abundant proteins in serum/plasma. This step allows us to deplete highly abundant albumin, IgG, transferrin, heptoglobin, IgM, IgA, fibrinogen, alpha antitrypsin, apolipoprotein A1, alpha 1 acid glycoprotein, alpha2 macroglobin, transthyretin, complement C3 and apolipoproteins. We had to optimize our procedures for depletion of abundant proteins from vitreous humor. For each depletion cycle, Agilent recommends 20 μl of serum that is approximately equivalent to 1 mg of total protein. During depletion of abundant proteins from vitreous, we also used 1 mg of protein for each depletion cycle. As we had 4 mg protein, we carried out 4 independent depletion cycles and pooled the flow through fractions. After passing the vitreous sample through MARS 14 column, 460 μg of protein was recovered in the flow through and the remaining 3.54 mg of protein accounted for the bound fraction. 60 μg of depleted vitreous sample was resolved on SDS-PAGE and subjected to in-gel digestion prior to mass spectrometry analysis. The remaining 400 μg of protein was reduced (5 mM DTT), alkylated (20 mM iodoacetamide) and digested using trypsin 1:20 w/w (Promega, Madison, WI) overnight at 37°C. The samples were acidified by adding 20% trifluoro acetic acid (TFA) to a final concentration of 0.1% and desalted using C_18_ macro-spin columns (Harvard apparatus, catalog no. 74-4101). 200 μg peptide digest was used for SCX fractionation as well as OFFGEL fractionation.

### In-gel digestion

Sixty micrograms of depleted vitreous sample was resolved by SDS-PAGE and stained using colloidal Coomassie stain. The lane was excised into 16 pieces and destained with 40 mM ammonium bicarbonate in 50% acetonitrile (ACN). Trypsin digestion was carried out essentially as described previously [66,67]. Briefly, reduction was carried out using 5 mM dithiothreitol (DTT, 60°C for 45 minutes) followed by alkylation using 20 mM iodoacetamide (room temperature for 10 min). Sequencing grade modified porcine trypsin (Promega, Madison, WI, US) in ammonium bicarbonate was added to the gel pieces at 4°C and incubated for 45 minutes. Excess trypsin was removed and the gel pieces were immersed in ammonium bicarbonate and incubated overnight at 37°C. The peptides were extracted from the gel bands using 0.4% formic acid in 3% ACN twice, once using 0.4% formic acid in 50% ACN and once using 100% ACN. The extracted peptides were dried using speedvac and stored at -80°C until LC-MS/MS analysis.

### Strong cation exchange chromatography

The peptide digest equivalent to 200 μg was reconstituted with 10 mM potassium phosphate buffer containing 30% ACN, pH 2.7 (solvent A). SCX fractionation was carried out using Polysulfoethyl A column (PolyLC, Columbia, MD) (300 Å, 5 μm, 100 × 2.1 mm) using an Agilent 1200 HPLC system (Agilent Technologies, Santa Clara, USA) containing a binary pump, UV detector and a fraction collector [68,69]. The peptides were eluted using a linear salt gradient (0 to 35%) of solvent B (10 mM potassium phosphate buffer containing 30% ACN, 350 mM KCl, pH 2.7) at a flow rate of 200 μl/min. The fractions were completely dried and reconstituted in 0.1% TFA. They were desalted using stage-tips and dried on speedvac.

### OFFGEL fractionation

The peptide digest equivalent to 200 μg in-solution digest was used for OFFGEL fractionation [70,71]. Agilent 3100 OFFGEL fractionator (Agilent Technologies, Santa Clara, USA) was used for pI based separation of peptides. As per the protocol, peptides were separated using pH 3-10 Immobilized pH gradient (IPG) strip, 13 cm. The peptides were focused at 50 kVh with maximum current of 50 μA and maximum voltage set to 4000 V. Twelve fractions were collected and acidified to obtain a final concentration of 0.1% TFA prior to sample cleaning using stage-tip protocol [72].

### LC-MS/MS analysis

LC-MS/MS analyses of the samples were carried out using high resolution Fourier transform mass spectrometer, LTQ-Orbitrap Velos (Thermo Electron, Bremen, Germany). The mass spectrometer was interfaced with a nano-LC system to a trap column (2 cm × 75 μm, C18 material 5 μm, 120 Å) and an analytical column (10 cm × 75 μm, C18 material 5 μm, 120 Å). Electrospray source was fitted with an 8 μm emitter tip (New Objective, Woburn, MA) and was applied a voltage of 2000 V. Peptide samples were loaded onto trap column in 3% solvent B (90% ACN in 0.1% formic acid) and washed for 5 minutes before peptide elution using a gradient of 3-35% solvent B for 60 minutes at a constant flow rate of 0.4 μl/min. Xcalibur 2.1 (Thermo Electron, Bremen, Germany) was used for data acquisition. The MS spectra were acquired in a data-dependent manner targeting the twenty most abundant ions in each survey scan in the range of m/z 350 to 1,800. Precursor ions selected for MS/MS fragmentation were dynamically excluded for 30s. Target ion quantity for FT full MS and MS2 were 5 × 10^5^ and 2 × 10^5^ respectively. Higher-energy collisional dissociation (HCD) was used for precursor fragmentation. MS and MS/MS data were acquired at a resolution of 60,000 and 15,000 at 400 m/z, respectively. Internal calibration was enabled using polydimethylcyclosiloxane (m/z, 445.1200025) ions and lock mass was used for accurate mass measurements.

### Data analysis

Mass spectrometry data was processed using the Proteome Discoverer software (Version 1.4.1.14, Thermo Fisher Scientific, Bremen, Germany). Mascot, SEQUEST and X! Tandem search engines were employed to maximize the peptide identification. The mass spectrometry data was searched against NCBI RefSeq 59 human protein database containing 36,211 sequences with known contaminants. Carbamidomethylation of cysteine was used as the fixed modification and oxidation of methionine and protein N-terminal acetylation as variable modifications. Peptide mass and fragment mass tolerance were set as 20 ppm and 0.1 Da, respectively with 1 missed cleavage. Peptide identifications were filtered by setting 1% target false discovery rate (FDR). Subcellular localization, molecular function and biological process of identified proteins were analyzed using gene ontology (GO) compliant databases - Human Protein Reference Database (HPRD: http://www.hprd.org) and Human Proteinpedia [63,73].

### Data availability

The raw data obtained from vitreous proteome are submitted to public data repositories. The peptide identifications and MS/MS spectra are available on Human Proteinpedia [63], (https://www.humanproteinpedia.org) as accession number HuPA_00682. The mass spectrometry proteomics data have been deposited to the ProteomeXchange Consortium [74] via the PRIDE partner repository with the dataset identifier PXD000957.

## Competing interest

All authors have expressed no conflict of interest.

## Authors’ contributions

KRM, PRM, HG, TSKP and AP conceptualized and designed the study. KRM and PRM provided the samples. KRM, RG and HKCJ contributed to sample preparation. NAS and RS performed the mass spectrometry experiments SMS, YS, AHP and A Parashar contributed to data analysis. KRM and RG wrote the manuscript. KRM, BGN, VK, TSKP, HG and AP designed the experiments, supervised the experiments and data analysis and critically reviewed the manuscript. All the authors have read and approved the final manuscript.

## Supplementary Material

Additional file 1: Table S1 A complete list of proteins identified in the vitreous humor. This table provides a list of all the proteins identified in vitreous including their gene symbol, RefSeq accession, protein description, number of peptides, sequence coverage, reported subcellular localization, molecular function, biological process and domains/motifs. Click here for file

Additional file 2: Table S2 A list of all peptides identified in the vitreous humor. Click here for file

## References

[B1] Jack RL Ultrastructure of the hyaloid vascular system Arch Ophthalmol 1972 11 5 555 567 5028097 10.1001/archopht.1972.01000020557014

[B2] Linsenmayer TF Gibney E Little CD Type II collagen in the early embryonic chick cornea and vitreous: immunoradiochemical evidence Exp Eye Res 1982 11 3 371 379 7067745 10.1016/0014-4835(82)90083-5

[B3] Walker F Patrick RS Constituent monosaccharides and hexosamine concentration of normal human vitreous humour Exp Eye Res 1967 11 3 227 232 6029413 10.1016/s0014-4835(67)80035-6

[B4] Locke JC Morton WR Further studies of the viscosity of aspirated human vitreous fluid: with special reference to its use in retinal detachment surgery Trans Am Ophthalmol Soc 1965 11 129 145 5859783 PMC1310189

[B5] Scott JE The chemical morphology of the vitreous Eye 1992 11 Pt 6 553 555 1289129 10.1038/eye.1992.120

[B6] Bishop PN Crossman MV McLeod D Ayad S Extraction and characterization of the tissue forms of collagen types II and IX from bovine vitreous Biochem J 1994 11 Pt 2 497 505 8172611 10.1042/bj2990497 PMC1138299

[B7] Ramakrishnan S Sulochana KN Parikh S Punitham R Transthyretin (prealbumin) in eye structures and variation of vitreous-transthyretin in diseases Indian J Ophthalmol 1999 11 1 31 34 16130282

[B8] Eichenbaum JW Zheng W Distribution of lead and transthyretin in human eyes J Toxicol Clin Toxicol 2000 11 4 377 381 10930053 10.1081/clt-100100946 PMC4988657

[B9] Clausen R Weller M Wiedemann P Heimann K Hilgers RD Zilles K An immunochemical quantitative analysis of the protein pattern in physiologic and pathologic vitreous Graefes Arch Clin Exp Ophthalmol 1991 11 2 186 190 2044983 10.1007/BF00170555

[B10] Jacobson B Identification of sialyl and galactosyl transferase activities in calf vitreous hyalocytes Curr Eye Res 1984 11 8 1033 1041 6091999 10.3109/02713688409011749

[B11] Yoshimura T Sonoda KH Sugahara M Mochizuki Y Enaida H Oshima Y Ueno A Hata Y Yoshida H Ishibashi T Comprehensive analysis of inflammatory immune mediators in vitreoretinal diseases PLoS One 2009 11 12 e8158 19997642 10.1371/journal.pone.0008158 PMC2780733

[B12] Nakanishi T Koyama R Ikeda T Shimizu A Catalogue of soluble proteins in the human vitreous humor: comparison between diabetic retinopathy and macular hole J Chromatogr B Analyt Technol Biomed Life Sci 2002 11 1 89 100 10.1016/s1570-0232(02)00078-8 12127329

[B13] Kim T Kim SJ Kim K Kang UB Lee C Park KS Yu HG Kim Y Profiling of vitreous proteomes from proliferative diabetic retinopathy and nondiabetic patients Proteomics 2007 11 22 4203 4215 17955474 10.1002/pmic.200700745

[B14] Gao BB Chen X Timothy N Aiello LP Feener EP Characterization of the vitreous proteome in diabetes without diabetic retinopathy and diabetes with proliferative diabetic retinopathy J Proteome Res 2008 11 6 2516 2525 18433156 10.1021/pr800112g

[B15] Ouchi M West K Crabb JW Kinoshita S Kamei M Proteomic analysis of vitreous from diabetic macular edema Exp Eye Res 2005 11 2 176 182 16080911 10.1016/j.exer.2005.01.020

[B16] Aretz S Krohne TU Kammerer K Warnken U Hotz-Wagenblatt A Bergmann M Stanzel BV Kemph T Holz FG Schnolzer M Kopitz J In-depth mass spectrometric mapping of the human vitreous proteome Proteome Sci 2013 11 1 22 23688336 10.1186/1477-5956-11-22 PMC3689628

[B17] Simo R Higuera M Garcia Ramirez M Canals F Garcia-Arumi J Hernandez C Elevation of apolipoprotein A-1 and apolipoprotein H levels in the vitreous fluid and overexpression in the retina of diabetic patients Arch Ophthalmol 2008 11 8 1076 1081 18695102 10.1001/archopht.126.8.1076

[B18] Wu CW Sauter JL Johnson PK Chen CD Olsen TW Identification and localization of major soluble proteins in human ocular tissue Am J Ophthalmol 2004 11 4 655 661 15059704 10.1016/j.ajo.2003.11.009

[B19] Yamane K Minamoto A Yamashita H Takamura H Miyamoto-Myoken Y Yoshizato K Nabetani T Tsugita A Mishima HK Proteome analysis of human vitreous proteins Mol Cell Proteomics 2003 11 11 1177 1187 12975481 10.1074/mcp.M300038-MCP200

[B20] Zhang J Gerhardinger C Lorenzi M Early complement activation and decreased levels of glycosylphosphatidylinositol-anchored complement inhibitors in human and experimental diabetic retinopathy Diabetes 2002 11 12 3499 3504 12453906 10.2337/diabetes.51.12.3499

[B21] Raychaudhuri S Iartchouk O Chin K Tan PL Tai AK Ripke S Gowrisankar S Vemuri S Montgomery K Yu Y Reynolds R Zack DJ Campochiaro B Campochiaro P Katsanis N Daly MJ Seddon JM A rare penetrant mutation in CFH confers high risk of age-related macular degeneration Nat Genet 2011 11 12 1232 1236 22019782 10.1038/ng.976 PMC3225644

[B22] Klein RJ Zeiss C Chew EY Tsai JY Sackler RS Haynes C Henning AK SanGiovanni JP Mane SM Mayne ST Bracken MB Ferris FL Ott J Barnstable C Hoh J Complement factor H polymorphism in age-related macular degeneration Science 2005 11 5720 385 389 15761122 10.1126/science.1109557 PMC1512523

[B23] Rodriguez de Cordoba S Esparza-Gordillo J Goicoechea de Jorge E Lopez-Trascasa M Sanchez-Corral P The human complement factor H: functional roles, genetic variations and disease associations Mol Immunol 2004 11 4 355 367 15163532 10.1016/j.molimm.2004.02.005

[B24] Ennis S Gibson J Cree AJ Collins A Lotery AJ Support for the involvement of complement factor I in age-related macular degeneration Eur J Hum Genet 2010 11 1 15 16 19603066 10.1038/ejhg.2009.113 PMC2987157

[B25] Reardon AJ Le Goff M Briggs MD McLeod D Sheehan JK Thornton DJ Bishop PN Identification in vitreous and molecular cloning of opticin, a novel member of the family of leucine-rich repeat proteins of the extracellular matrix J Biol Chem 2000 11 3 2123 2129 10636917 10.1074/jbc.275.3.2123

[B26] Ramesh S Bonshek RE Bishop PN Immunolocalisation of opticin in the human eye Br J Ophthalmol 2004 11 5 697 702 15090426 10.1136/bjo.2003.031989 PMC1772128

[B27] Friedman JS Faucher M Hiscott P Biron VL Malenfant M Turcotte P Raymond V Walter MA Protein localization in the human eye and genetic screen of opticin Hum Mol Genet 2002 11 11 1333 1342 12019215 10.1093/hmg/11.11.1333

[B28] Friedman JS Ducharme R Raymond V Walter MA Isolation of a novel iris-specific and leucine-rich repeat protein (oculoglycan) using differential selection Invest Ophthalmol Vis Sci 2000 11 8 2059 2066 10892843

[B29] Liou GI Ma DP Yang YW Geng L Zhu C Baehr W Human interstitial retinoid-binding protein. Gene structure and primary structure J Biol Chem 1989 11 14 8200 8206 2542268

[B30] Taniguchi T Adler AJ Mizuochi T Kochibe N Kobata A The structures of the asparagine-linked sugar chains of bovine interphotoreceptor retinol-binding protein. Occurrence of fucosylated hybrid-type oligosaccharides J Biol Chem 1986 11 4 1730 1736 3944106

[B31] Hollyfield JG Fliesler SJ Rayborn ME Bridges CD Rod photoreceptors in the human retina synthesize and secrete interstitial retinol-binding protein Prog Clin Biol Res 1985 11 141 149 4048219

[B32] Barnstable CJ Tombran-Tink J Neuroprotective and antiangiogenic actions of PEDF in the eye: molecular targets and therapeutic potential Prog Retin Eye Res 2004 11 5 561 577 15302351 10.1016/j.preteyeres.2004.05.002

[B33] Pepperberg DR Okajima TL Wiggert B Ripps H Crouch RK Chader GJ Interphotoreceptor retinoid-binding protein (IRBP). Molecular biology and physiological role in the visual cycle of rhodopsin Mol Neurobiol 1993 11 1 61 85 8318167 10.1007/BF02780609

[B34] Den Hollander AI McGee TL Ziviello C Banfi S Dryja TP Gonzalez-Fernandez F Ghosh D Berson EL A homozygous missense mutation in the IRBP gene (RBP3) associated with autosomal recessive retinitis pigmentosa Invest Ophthalmol Vis Sci 2009 11 4 1864 1872 19074801 10.1167/iovs.08-2497 PMC2823395

[B35] Foltz DR Nye JS Hyperphosphorylation and association with RBP of the intracellular domain of Notch1 Biochem Biophys Res Commun 2001 11 3 484 492 11511084 10.1006/bbrc.2001.5421

[B36] Ortego J Escribano J Becerra SP Coca-Prados M Gene expression of the neurotrophic pigment epithelium-derived factor in the human ciliary epithelium. Synthesis and secretion into the aqueous humor Invest Ophthalmol Vis Sci 1996 11 13 2759 2767 8977492

[B37] Meyer C Notari L Becerra SP Mapping the type I collagen-binding site on pigment epithelium-derived factor. Implications for its antiangiogenic activity J Biol Chem 2002 11 47 45400 45407 12237317 10.1074/jbc.M208339200

[B38] Fan W Crawford R Xiao Y The ratio of VEGF/PEDF expression in bone marrow mesenchymal stem cells regulates neovascularization Differentiation 2011 11 3 181 191 21236558 10.1016/j.diff.2010.12.003

[B39] Park K Jin J Hu Y Zhou K Ma JX Overexpression of pigment epithelium-derived factor inhibits retinal inflammation and neovascularization Am J Pathol 2011 11 2 688 698 21281801 10.1016/j.ajpath.2010.10.014 PMC3070578

[B40] Garcia-Ramirez M Canals F Hernandez C Colome N Ferrer C Carrasco E Garcia-Arumi J Simo R Proteomic analysis of human vitreous fluid by fluorescence-based difference gel electrophoresis (DIGE): a new strategy for identifying potential candidates in the pathogenesis of proliferative diabetic retinopathy Diabetologia 2007 11 6 1294 1303 17380318 10.1007/s00125-007-0627-y

[B41] Noma H Funatsu H Mimura T Eguchi S Shimada K Hori S Vitreous levels of pigment epithelium-derived factor and vascular endothelial growth factor in macular edema with central retinal vein occlusion Curr Eye Res 2011 11 3 256 263 21275514 10.3109/02713683.2010.513090

[B42] Konson A Pradeep S D'Acunto CW Seger R Pigment epithelium-derived factor and its phosphomimetic mutant induce JNK-dependent apoptosis and p38-mediated migration arrest J Biol Chem 2011 11 5 3540 3551 21059648 10.1074/jbc.M110.151548 PMC3030359

[B43] Khanna AK Meher S Prakash S Tiwary SK Singh U Srivastava A Dixit VK Comparison of Ranson, Glasgow, MOSS, SIRS, BISAP, Apache-II, CTSI scores, IL-6, CRP and Procalcitonin in predicting severity, organ failure, pancreatic necrosis and mortality in acute pancratitis HPB Surg 2013 11 367581 24204087 10.1155/2013/367581 PMC3800571

[B44] Ucmak D Akkurt M Toprak G Yesilova Y Turan E Yıldız I Determination of dermatology life quality index, and serum C-reactive protein and plasma interleukin-6 levels in patients with chronic urticaria Postepy Dermatol Alergol 2013 11 3 146 151 24278066 10.5114/pdia.2013.35615 PMC3834718

[B45] Clemmons DR Role of insulin-like growth factor binding proteins in controlling IGF actions Mol Cell Endocrinol 1998 11 1–2 19 24 9722163 10.1016/s0303-7207(98)00024-0

[B46] Clemmons DR Insulin-like growth factor binding proteins and their role in controlling IGF actions Cytokine Growth Factor Rev 1997 11 1 45 62 9174662 10.1016/s1359-6101(96)00053-6

[B47] Clemmons DR Busby W Clarke JB Parker A Duan C Nam TJ Modifications of insulin-like growth factor binding proteins and their role in controlling IGF actions Endocr J 1998 11 Suppl S1 S8 9790222 10.1507/endocrj.45.suppl_s1

[B48] Ceda GP Fielder PJ Henzel WJ Louie A Donovan SM Hoffman AR Rosenfeld RG Differential effects of insulin-like growth factor (IGF)-I and IGF-II on the expression of IGF binding proteins (IGFBPs) in a rat neuroblastoma cell line: isolation and characterization of two forms of IGFBP-4 Endocrinology 1991 11 6 2815 2824 1709857 10.1210/endo-128-6-2815

[B49] Shimasaki S Gao L Shimonaka M Ling N Isolation and molecular cloning of insulin-like growth factor-binding protein-6 Mol Endocrinol 1991 11 7 938 948 1719383 10.1210/mend-5-7-938

[B50] Li Z Picard F Modulation of IGFBP2 mRNA expression in white adipose tissue upon aging and obesity Horm Metab Res 2010 11 11 787 791 20730703 10.1055/s-0030-1262854

[B51] Koyama N Zhang J Huqun Miyazawa H Tanaka T Su X Hagiwara K Identification of IGFBP-6 as an effector of the tumor suppressor activity of SEMA3B Oncogene 2008 11 51 6581 6589 18985860 10.1038/onc.2008.263

[B52] Ehrenborg E Zazzi H Lagercrantz S Granqvist M Hillerbrand U Allander SV Larsson C Luthman H Characterization and chromosomal localization of the human insulin-like growth factor-binding protein 6 gene Mamm Genome 1999 11 4 376 380 10087296 10.1007/s003359901005

[B53] Neumann GM Marinaro JA Bach LA Identification of O-glycosylation sites and partial characterization of carbohydrate structure and disulfide linkages of human insulin-like growth factor binding protein 6 Biochemistry 1998 11 18 6572 6585 9572875 10.1021/bi972894e

[B54] Migita T Narita T Asaka R Miyagi E Nagano H Nomura K Matsuura M Satoh Y Okumura S Nakagawa K Seimiya H Ishikawa Y Role of insulin-like growth factor binding protein 2 in lung adenocarcinoma: IGF-independent antiapoptotic effect via caspase-3 Am J Pathol 2010 11 4 1756 1766 20150439 10.2353/ajpath.2010.090500 PMC2843467

[B55] Zhuang T Chen Q Cho MK Vishnivetskiy SA Iverson TM Gurevich VV Sanders CR Involvement of distinct arrestin-1 elements in binding to different functional forms of rhodopsin Proc Natl Acad Sci U S A 2013 11 3 942 947 23277586 10.1073/pnas.1215176110 PMC3549108

[B56] Zhao C Yang P He H Lin X Li B Zhou H Huang X Kijlstra A S-antigen specific T helper type 1 response is present in Behcet's disease Mol Vis 2008 11 1456 1464 18685727 PMC2496927

[B57] Huang L Li W Tang W Zhu X Ou-Yang P Lu G A Chinese family with Oguchi’s disease due to compound heterozygosity including a novel deletion in the arrestin gene Mol Vis 2012 11 528 536 22419846 PMC3298420

[B58] Fujinami K Tsunoda K Nakamura M Oguchi Y Miyake Y Oguchi disease with unusual findings associated with a heterozygous mutation in the SAG gene Arch Ophthalmol 2011 11 10 1375 1376 21987685 10.1001/archophthalmol.2011.300

[B59] Fuchs S Nakazawa M Maw M Tamai M Oguchi Y Gal A A homozygous 1-base pair deletion in the arrestin gene is a frequent cause of Oguchi disease in Japanese Nat Genet 1995 11 3 360 362 7670478 10.1038/ng0795-360

[B60] Shi Y Massagué J Mechanisms of TGF-beta signaling from cell membrane to the nucleus Cell 2003 11 6 685 700 12809600 10.1016/s0092-8674(03)00432-x

[B61] Wordinger RJ Fleenor DL Hellberg PE Pang IH Tovar TO Zode GS Fuller JA Clark AF Effects of TGF-beta2, BMP-4, and gremlin in the trabecular meshwork: implications for glaucoma Invest Ophthalmol Vis Sci 2007 11 3 1191 1200 17325163 10.1167/iovs.06-0296

[B62] David D Cardoso J Marques B Marques R Silva ED Santos H Boavida MG Molecular characterization of a familial translocation implicates disruption of HDAC9 and possible position effect on TGFbeta2 in the pathogenesis of Peters’ anomaly Genomics 2003 11 5 489 503 12706107 10.1016/s0888-7543(03)00046-6

[B63] Mathivanan S Ahmed M Ahn NG Alexandre H Amanchy R Andrews PC Bader JS Balgley BM Bantscheff M Bennett KL Bjorling E Blagoev B Bose R Brahmachari SK Burlingame AS Bustelo XR Cagney G Cantin GT Cardasis HL Celis JE Chaerkady R Chu F Cole PA Costello CE Cotter RJ Crockett D DeLany JP De Marzo AM DeSouza LV Deutsch EW Human Proteinpedia enables sharing of human protein data Nat Biotechnol 2008 11 2 164 167 18259167 10.1038/nbt0208-164

[B64] Mishra GR Suresh M Kumaran K Kannabiran N Suresh S Bala P Shivakumar K Anuradha N Reddy R Raghavan TM Menon S Hanumanthu G Gupta M Upendran S Gupta S Mahesh M Jacob B Mathew P Chatterjee P Arun KS Sharma S Chandrika KN Deshpande N Palvankar K Raghavnath R Krishnakanth R Karathia H Rekha B Nayak R Vishnupriya G Human protein reference database–2006 update Nucleic Acids Res 2006 11 Database issue D411 D414 16381900 10.1093/nar/gkj141 PMC1347503

[B65] Goel R Harsha HC Pandey A Prasad TS Human Protein Reference Database and Human Proteinpedia as resources for phosphoproteome analysis Mol Biosyst 2012 11 2 453 463 22159132 10.1039/c1mb05340j PMC3804167

[B66] Harsha HC Molina H Pandey A Quantitative proteomics using stable isotope labeling with amino acids in cell culture Nat Protoc 2008 11 3 505 516 18323819 10.1038/nprot.2008.2

[B67] Goel R Murthy KR Srikanth SM Pinto SM Bhattacharjee M Kelkar DS Madugundu AK Dey G Mohan SS Venkatarangaiah K Prasad TSK Chakravarti S Harsha HC Pandey A Characterizing the normal proteome of human ciliary body Clin Proteomics 2013 11 1 9 23914977 10.1186/1559-0275-10-9 PMC3750387

[B68] Chaerkady R Harsha HC Nalli A Gucek M Vivekanandan P Akhtar J Cole RN Simmers J Schulick RD Singh S Torbenson M Pandey A Thuluvath PJ A quantitative proteomic approach for identification of potential biomarkers in hepatocellular carcinoma J Proteome Res 2008 11 10 4289 4298 18715028 10.1021/pr800197z PMC3769105

[B69] Yang Y Chaerkady R Kandasamy K Huang TC Selvan LD Dwivedi SB Kent OA Mendell JT Pandey A Identifying targets of miR-143 using a SILAC-based proteomic approach Mol Biosyst 2010 11 10 1873 1882 20544124 10.1039/c004401f PMC3812686

[B70] Barbhuiya MA Sahasrabuddhe NA Pinto SM Mutjusamy B Singh TD Nanjappa V Keerthikumar S Delanghe B Harsha HC Chaerkady R Jalaj V Gupta S Srivastav BR Tiwari PK Pandey A Comprehensive proteomic analysis of human bile Proteomics 2011 11 23 4443 4453 22114102 10.1002/pmic.201100197

[B71] Kelkar DS Kumar D Kumar P Muthusamy B Yadav AK Srivastava P Marimuthu A Anand S Sundaram H Kingsbury R Harsha HC Nair B Prasad TS Chauhan DS Katoch K Katoch VM Kumar P Chaerkady R Ramachandran S Dash D Pandey A Proteogenomic analysis of Mycobacterium tuberculosis by high resolution mass spectrometry Mol Cell Proteomics 2011 11 12 M111.011627 doi: 10.1074/mcp.M111.011445. Epub 2011 Oct 3 21969609 10.1074/mcp.M111.011627 PMC3275902

[B72] Rappsilber J Mann M Ishihama Y Protocol for micro-purification, enrichment, pre-fractionation and storage of peptides for proteomics using StageTips Nat Protoc 2007 11 8 1896 1906 17703201 10.1038/nprot.2007.261

[B73] Keshava Prasad TS Goel R Kandasamy K Keerthikumar S Kumar S Mathivanan S Telikicherla D Raju R Shafreen B Venugopal A Balakrishnan L Marimuthu A Banerjee S Somanathan DS Sebastian A Rani S Ray S Harrys Kishore CJ Kanth S Ahmed M Kashyap MK Mohmood R Ramachandra YL Krishna V Rahiman BA Mohan S Ranganathan P Ramabadran S Chaerkady R Pandey A Human Protein Reference Database--2009 update Nucleic Acids Res 2009 11 Database issue D767 D772 18988627 10.1093/nar/gkn892 PMC2686490

[B74] Vizcaíno JA Deutsch EW Wang R Csordas A Reisinger F Ríos D Dianes JA Sun Z Farrah T Bandeira N Binz PA Xenarios I Eisenacher M Mayer G Gatto L Campos A Chalkley RJ Kraus HJ Albar JP Martinez-Bartolomé S Apweiler R Omenn GS Martens L Jones AR Hermjakob H ProteomeXchange provides globally co-ordinated proteomics data submission and dissemination Nature Biotechnol 2014 11 3 223 226 24727771 10.1038/nbt.2839 PMC3986813

